# Single-Cell Genomics Uncovers an Unexpectedly Abundant, Phylogenetically Hyperdiverse Patescibacteria (Candidate Phyla Radiation) Community in Agricultural Paddy Soil

**DOI:** 10.3390/microorganisms14102213

**Published:** 2026-10-02

**Authors:** Arslan Ali, Daisuke Saisho, Keiichi Mochida, Akio Tani

**Affiliations:** 1 Institute of Plant Science and Resources, Okayama University, Kurashiki 710-0046, Okayama, Japan; p1fv5iv1@s.okayama-u.ac.jp (A.A.); saisho@okayama-u.ac.jp (D.S.); 2 RIKEN Center for Sustainable Resource Science, Yokohama 230-0045, Kanagawa, Japan

**Keywords:** Patescibacteria, Candidate Phyla Radiation (CPR), single-cell genomics, paddy soil

## Abstract

Patescibacteria, also known as the Candidate Phyla Radiation (CPR), are among the most diverse yet functionally reduced bacterial lineages whose diversity in agricultural soils remains poorly characterized. We applied single-cell genomics to bulk soil from an unfertilized paddy field after rice harvest and drainage. From 384 randomly sorted single cells, 126 medium-to-high-quality SAGs (Single-Amplified Genomes) were recovered, spanning 24 bacterial and archaeal phyla. Unexpectedly, 108 of the 384 SAGs (28.1%) were assigned to Patescibacteria—the most frequently recovered phylum—including 42 of the 126 reliable SAGs. These genomes were notably small (mean 973 kb vs. 1.4–3.2 Mb for other phyla). Because CheckM underestimates completeness in reduced CPR genomes, CheckM2 was applied, raising mean completeness from 59.9% to 83.3% and reclassifying 15 genomes as near-complete (>90%). Phylogenomic analysis using 16 ribosomal protein markers revealed extensive diversity and low average amino acid identity to reference genomes (mean 44.6%), indicating substantial taxonomic novelty. Despite this diversity, functional annotation and OrthoFinder analysis showed a conserved, reduced gene repertoire dominated by translation and DNA repair genes and depleted in amino acid, cofactor, and nucleotide biosynthesis. These results show that modest soil sampling can recover outsized Patescibacteria diversity, underscoring the need for CPR-appropriate completeness assessment.

## 1. Introduction

Microbial dark matter makes up a significant portion of microbial diversity that remains poorly characterized owing to low cultivation success [1]. The Candidate Phyla Radiation (CPR), or Patescibacteria, is an extensive and deeply branching bacterial lineage known almost exclusively through cultivation-independent techniques, including metagenomics and single-cell genomics (SCG) [2]. Members of Patescibacteria generally have ultra-small cells, highly streamlined genomes with a reduced complement of biosynthetic pathways, and unusual ribosomal and metabolic features that distinguish them from well-studied bacterial groups [3]. These characteristics have made them difficult to study by conventional culture-based techniques, so most of their known diversity comes from sequence-based reconstruction [4].

Patescibacteria genome streamlining is thought to reflect low metabolic potential and a symbiotic or host-associated lifestyle, though free-living activity has also been proposed in some settings [5]. Their reduced genomes typically lack central metabolic and amino acid biosynthesis pathways, restricting independent growth in niches where metabolites are obtained from other organisms or the environment [6]; genome streamlining is documented across environments, with groundwater CPR taxa tending toward even more compact genomes than soil counterparts [7]. Patescibacteria metagenome-assembled genomes show near-complete loss of de novo biosynthetic capacity for amino acids, nucleotides, fatty acids, and cofactors [8,9], while commonly retaining a partial glycolytic pathway and pyruvate-converting enzymes that enable substrate-level phosphorylation without a complete TCA cycle or electron transport chain [10]. Rather than biochemical self-sufficiency, Patescibacteria are enriched in proteases, oligopeptide transporters, nucleases, and ABC transporters consistent with an episymbiotic, metabolite-scavenging lifestyle [11], and several lineages encode type IV pili and adhesion proteins for physical symbiosis, as exemplified by the two distinct pilus systems the cultivated strain Saccharibacteria TM7x uses for host attachment and motility [12].

Despite this low autonomous metabolism, Patescibacteria are ubiquitous across subsurface, aquatic, soil, and host-associated ecosystems and can shape microbial community composition. Groundwater metatranscriptomes show active ribosome production and DNA-repair expression alongside near-absent biosynthetic transcripts, consistent with a metabolite-scavenging, replicative-symbiont lifestyle [13], and Patescibacteria can dominate subsurface assemblages, exceeding 66% of the community in some cases [14]; elevated abundances in seepage water further suggest soils may be an important source of CPR taxa entering subsurface systems [15].

Agricultural soils are among the most actively managed terrestrial biomes on Earth, yet they remain comparatively underrepresented in genome-resolved studies of Patescibacteria. Amplicon sequencing and bulk metagenomics often fail to recover CPR clades at low relative abundance or cannot reconstruct high-quality genomes for them, owing to the physical and chemical complexity of the soil matrix and the extremely small size of these cells [7]; conventional metagenome assembly and binning are also biased against ultra-small genomes and low-coverage scaffolds, which can lead to underestimation of CPR diversity and genomic potential. This gap is significant because agricultural soils, subject to repeated disturbance and nutrient input, could host Patescibacteria lineages with distinctive ecological roles in nutrient cycling and soil health. Prior work has shown that CPR populations in soil seepage and groundwater vary considerably with environmental context [15], but comparable genome-resolved characterization has been lacking for agricultural soils specifically, and paddy soils—subject to a recurring cycle of flooding and drainage that repeatedly reshapes redox conditions, carbon availability, and microbial community structure—represent a particularly understudied and dynamic case within this broader gap.

SCG is well suited to closing this gap because it recovers genomes from individual cells without the enrichment-culturing biases of cultivation-based approaches or the assembly/binning biases that affect low-abundance or highly divergent lineages in shotgun metagenomics [16]. By combining fluorescence-activated cell sorting with whole-genome amplification and careful downstream assembly and taxonomic assignment, SCG can produce genome assemblies for rare or hard-to-assemble taxa such as ultra-small Patescibacteria that are underrepresented in metagenomic data [17]. This approach has previously been applied successfully in groundwater and other aquatic systems, recovering CPR genomes that expand phylogenetic coverage and reveal lineage-specific phenotypes not visible in bulk metagenomes [16,18], but it has not, to our knowledge, previously been extended to agricultural soils, where management practices, nutrient fluxes, and soil microbial networks may shape CPR composition in distinctive ways.

The present study did not originally set out to specifically target Patescibacteria. As part of a broader effort to characterize root-associated versus bulk-soil microbiomes in a rice paddy field, we performed random, non-selective single-cell sorting and sequencing of bulk soil from an unfertilized experimental plot, collected shortly after rice harvest with the field water already drained. This sample was intended as an unbiased baseline snapshot of the soil community prior to any root-selection. Unexpectedly, once genomes of reliable quality were identified, Patescibacteria proved to be the single most heavily represented phylum in the dataset—prompting the more detailed, genome-resolved characterization presented here. Using this unbiased single-cell dataset, our goals were to: (i) determine whether the modest completeness scores returned by standard genome-quality tools for these Single-Amplified Genomes (SAGs) reflect true assembly incompleteness or an artifact of the marker genes those tools rely on; (ii) place the recovered Patescibacteria population—both by genome-resolved phylogenomics and by 16S rRNA gene comparison—within the context of previously described diversity in the phylum, including how agricultural/paddy soil-derived SAGs compare with CPR populations from other, better-studied ecosystems such as groundwater; and (iii) characterize the gene content of these genomes to assess their metabolic capacity relative to the limited existing characterization of CPR from other soils. We present this as an exploratory, single-sample characterization, and set a genome-resolved benchmark for Patescibacteria in agricultural soils that underscores the value of SCG for revealing latent soil microbial diversity.

## 2. Materials and Methods

### 2.1. Sample Collection

Soil was collected on 26 September 2022 from a non-fertilized experimental paddy plot at the Institute of Plant Science and Resources (IPSR), Okayama University, Kurashiki, Okayama, Japan (34°35′32″ N, 133°46′10″ E). The experimental plot has been used for double cropping of rice and barley, maintained under long-term (>25 years) non-fertilized management, allowing the development of a soil microbial community under nutrient-limited conditions. Soil was sampled from the 7 to 8 cm depth horizon of the topsoil following rice harvest and after the paddy field water had already been drained. The soil was homogenized prior to downstream processing. This bulk-soil sample served as the unselected baseline for a broader study comparing bulk-soil and root-associated (rice and barley cropping practiced in the institute) microbiomes in the same experimental field; no additional replicates, different timepoints or fertilized-field soil samples were analyzed in the present study.

### 2.2. Single-Cell Isolation, Whole-Genome Amplification, and Sequencing

The soil sample was subjected to single-cell genome analysis using bit-MAP^®^ technology (bitBiome Inc., Tokyo, Japan). Briefly, soil microbial cells detached from soil particles were encapsulated in gel microdroplets, lysed in situ, and subjected to whole-genome amplification (WGA) within the droplets [19]. Droplets showing successful amplification were sorted by flow cytometry into sterile microplates, and a total of 384 single cells were selected and carried through library preparation and short-read Illumina sequencing using an Illumina NextSeq 2000 system (Illumina, Inc., San Diego, CA, USA) based on the SAG-gel workflow, as previously described [20] (Supplementary File S1).

### 2.3. Read Processing and Decontamination

Raw sequencing reads were quality- and adapter-trimmed with BBTools bbduk.sh v38.90 [21] (ktrim = r, ref = adapters, k = 23, mink = 11, hdist = 1, tpe, tbo, qtrim = r, trimq = 10, minlength = 40, maxns = 1, minavgquality = 15). To remove potential human or laboratory contamination, quality-filtered reads were mapped against a masked human reference genome (hg19_main_mask_ribo_animal_allplant_allfungus.fa.gz) using bbmap.sh v38.90 (quickmatch, fast, untrim, minid = 0.95, maxindel = 3, bwr = 0.16, bw = 12, minhits = 2, qtrim = rl, trimq = 10); reads mapping to the human genome were classified as contaminants and excluded, and only unmapped reads were retained for assembly [21].

### 2.4. Single-Cell Genome Assembly and Annotation

Decontaminated reads from every single cell were assembled independently with SPAdes v3.15.2 in single-cell mode [22] (--sc --careful --disable-rr --disable-gzip-output, k = 21, 33, 55, 77, 99, 127, to improve recovery of fragmented single-cell genomes). Contigs shorter than 200 bp were removed with seqkit v0.12.1 [23]. Gene prediction and functional annotation, including Clusters of Orthologous Genes (COG) assignment, were performed with Prokka v1.14.5 [24] (--rawproduct --mincontiglen 200), ensuring standardized annotation across all SAGs. Assembly statistics (contig number, total assembly length, GC content, N50, etc.) were computed with QUAST v5.0.2 using default parameters [25].

### 2.5. Genome Quality Assessment and Taxonomic Classification

Genome completeness and contamination were estimated with CheckM v1.1.3 [26] (lineage_wf -r --nt; taxonomy_wf --nt, domain Bacteria). Genome quality tiers followed MIMAG-like conventions [27]: High quality, completeness >90%, contamination <5%, with 5S/16S/23S rRNA genes detected and ≥18 tRNA types identified; Medium quality, completeness ≥50%, contamination <10%; Low quality, completeness <50%; Contaminated, contamination ≥10%. Of the 384 total SAGs, 126 (2 High- and 124 Medium-quality) were retained as reliable for downstream analysis, while 212 were Low quality and 46 were Contaminated. Taxonomic classification was performed for all 384 SAGs with GTDB-Tk v2.1.0 [28] against reference database release 207_v2, assigning 108 SAGs (28.1% of the total dataset) to the phylum Patescibacteria; of these, 42 met Medium-quality thresholds and form the focal dataset for the genome-resolved analyses described below (the remaining 66—55 Low-quality and 11 Contaminated—were excluded from deeper comparative genomics but retained in the broader 16S rRNA survey, Section 2.14).

### 2.6. CheckM2 Reassessment of Patescibacteria SAGs

Because CheckM’s lineage-specific completeness estimate depends on a universal single-copy marker set dominated by ribosomal proteins and other translational components that are frequently reduced or divergent in CPR lineages, the 42 Patescibacteria SAGs were additionally reassessed for completeness and contamination using CheckM2 v1.1.0 [29], which estimates completeness from a machine-learning model trained on genome-wide sequence composition rather than a fixed marker set, using its DIAMOND-based reference database (uniref100.KO).

### 2.7. Reference Genome Retrieval

For each of the 42 SAGs, the closest publicly available NCBI assembly, identified via an average nucleotide identity (ANI)/alignment-fraction-based novelty screen, was noted; five such reference accessions were represented among the 42 SAGs (GCA_016196605.1, GCA_001000035.1, GCA_903834855.1, GCA_016215405.1, GCA_002772455.1) and were downloaded via the NCBI Datasets v2 API. GCA_903834855.1 lacked an existing protein annotation in the downloaded dataset and was annotated de novo with Prodigal v2.6.3 in metagenome mode [30].

### 2.8. 16S rRNA Gene Recovery and Phylogenetic Analysis

All 108 SAGs assigned to Patescibacteria were screened for 16S rRNA genes using ContEst16S (EzBioCloud) to further verify taxonomic assignment [31]. Owing to the fragmented and variably complete nature of single-cell assemblies, near-full-length 16S rRNA sequences were not recovered from every genome: 58 of the 108 Patescibacteria SAGs yielded near-full-length 16S rRNA sequences suitable for phylogenetic analysis. These were classified against the SILVA 138.2 SSURef NR99 reference database, aligned with MAFFT v7.520 (default parameters) [32], and used to infer a maximum-likelihood phylogeny with IQ-TREE v2.3 using automatic model selection and ultrafast bootstrap approximation [33]. The resulting tree was visualized in the Interactive Tree of Life (iTOL) v7.6, with sequences recovered in this study highlighted by colored outer stripes indicating phylum-level taxonomy, enabling direct comparison between agricultural soil-derived Patescibacteria SAGs and reference sequences from other habitats.

As a separate, finer-scale comparison restricted to the 42 medium/high-quality Patescibacteria SAGs used in genome-resolved analyses, 16S rRNA gene sequences were additionally identified from the existing Prokka annotation (feature type rRNA, product containing “16S”) and extracted from the corresponding nucleotide transcript files, retaining the longest copy where more than one was annotated. Because the five NCBI reference genomes lacked an equivalent rRNA annotation, their 16S rRNA genes were predicted de novo with barrnap v0.9 (bacterial mode, default parameters); one of the five references had no detectable 16S gene and was excluded from this comparison. All recovered 16S sequences (32 SAGs plus 4 references, 36 sequences total) were aligned with MAFFT v7 (--auto), and pairwise percent identity was calculated directly from the alignment for each genome pair, counting only alignment columns without gaps in either sequence and requiring at least 200 bp of mutual overlap.

### 2.9. Phylogenomic Analysis

Sixteen universal ribosomal protein marker genes (the rp16 set: uL2, uL3, uL4, uL5, uL6, uL14, uL15, uL16, uL18, uL22, uL24, uS3, uS8, uS10, uS17, uS19; Pfam accessions PF00181, PF00297, PF00573, PF00281, PF00347, PF00238, PF00828, PF00252, PF00861, PF00237, PF17136, PF00189, PF00410, PF00338, PF00366, PF00203) were identified in each genome’s predicted proteome with hmmsearch (HMMER v3 [34]; E < 1 × 10^−5^), retaining the single best-scoring hit per marker per genome. Genomes with fewer than 8 of the 16 markers were excluded from tree construction, retaining 35 of the 42 SAGs and 4 of the 5 reference genomes (GCA_016196605.1 was excluded from the tree for retaining only 6/16 markers but was kept in pairwise AAI comparisons). Each marker was aligned across retained genomes with MAFFT v7 (--auto) and trimmed with trimAl (-automated1 [35]). Trimmed alignments were concatenated into a single supermatrix (2040 amino acids for the 39-taxon alignment including reference genomes), with per-marker partitions retained. Maximum-likelihood phylogenies were inferred with IQ-TREE2 [33] under a partitioned substitution model selected with ModelFinder (-m TESTMERGE) and 1000 ultrafast bootstrap replicates.

### 2.10. Genome Similarity: Average Nucleotide and Amino Acid Identity

Pairwise average nucleotide identity (ANI) among the 42 SAGs and 5 reference genome assemblies was computed with fastANI v1.34 [36] using default parameters, all genomes against all genomes. Average amino acid identity (AAI) was computed as a reciprocal-best-hit average: predicted proteomes of all 47 genomes (45,771 proteins total) were combined and searched all-vs-all with DIAMOND blastp [37,38] (E < 1 × 10^−5^). For each genome pair, a protein pair was retained as a reciprocal best hit if each protein’s single highest-bitscore hit in the partner genome was, in turn, the query protein itself; AAI for that pair was the mean percent identity across all such reciprocal-best-hit protein pairs.

### 2.11. Functional Annotation (COG/KEGG)

COG category composition was derived from the COG identifiers assigned during Prokka annotation, mapped to functional categories using the NCBI COG database definition files (cog-20.def.tab and fun-20.tab, 2020 release). KEGG Ortholog (KO) assignment was performed with KofamScan v1.3.0 [39] against all predicted proteins of the 42 SAGs and the four reference genomes retained in the rp16 tree, using the KOfam HMM profile database and associated score thresholds. KEGG pathway and module completeness fractions were computed from the resulting KO annotations with KEGG-Decoder [40].

### 2.12. Orthology-Based Comparative Genomics

Predicted proteins from all 42 SAGs were clustered into orthogroups with OrthoFinder v3.1.5 [41], run with default settings (DIAMOND all-vs-all search, MCL clustering, gene tree/species tree inference). Orthogroups were classified as core (present in ≥90% of the 42 genomes), shell (10–90%), or cloud (<10%, including genome-specific singletons). For the 70 most broadly shared orthogroups (each detected in at least 30 of the 42 genomes), member genes were cross-referenced by locus tag against the existing Prokka product and COG annotations to assign a representative functional label and broad functional category (Supplementary Figure S1).

### 2.13. Identification of Virus-Dominated Single-Amplified Genomes

Because the single-cell sorting workflow was performed without prior taxonomic selection, non-cellular DNA, including free viral particles, could also be recovered during whole-genome amplification and sorting. To identify potential virus-dominated SAGs, all assemblies were analyzed using geNomad v1.12.0 [42] with default parameters. Candidate virus-derived SAGs were identified based on the proportion of virus-classified contigs relative to total assembly length, together with CheckM completeness and GTDB-Tk taxonomic assignments. SAGs in which virus-classified contigs represented at least 50% of the assembled genome were considered candidate virus-particle captures and were subjected to additional characterization. Because viral genome analysis was not the primary objective of this study, only a summary of these findings is presented, while detailed analyses are provided in the Supplementary Table S3.

### 2.14. Analysis of “Contaminated” SAGs

SAGs with CheckM contamination estimates of at least 10% were classified as Contaminated according to MIMAG-like criteria. To determine whether elevated contamination estimates reflected genuine mixing of DNA from different organisms, the 11 Patescibacteria-classified SAGs tagged as “Contaminated” were analyzed separately. The 16S rRNA genes were independently predicted from their assembled contigs using barrnap v0.9. Where multiple 16S rRNA gene copies were detected within the same SAG, the sequences were aligned using MAFFT and pairwise identities were calculated over aligned, non-gap positions. Individual 16S sequences were taxonomically classified against the SILVA 138.2 reference database using QIIME 2 [43], allowing their classifications to be compared with the genome-wide GTDB-Tk assignment. Detailed procedures, including the composition-based analysis and targeted examination of SAG 220926_sc-00194, are provided in the Supplementary Table S4.

### 2.15. Data Availability

Raw sequencing reads generated in this study have been submitted to the DNA Data Bank of Japan (DDBJ) Sequence Read Archive (DRA) under BioProject accession PRJDB42729. Each of the 384 single-amplified genomes (SAGs) was registered as an individual BioSample (accession numbers SAMD01947188–SAMD01947454 and SAMD01949276–SAMD01949392) with a corresponding individual DRA Run accession (DRR1079629–DRR1080012); the full SAG-to-BioSample-to-Run correspondence is provided in Supplementary Table S1. Genome quality statistics, taxonomic assignments, geNomad predictions, and 16S rRNA classifications, KEGG pathway annotation outputs, COG functional category assignments, and pairwise average amino acid identity (AAI) matrices are provided in Supplementary Tables S1–S7, respectively.

## 3. Results

### 3.1. Recovery, Taxonomic Composition, and Genome Characteristics of Patescibacteria SAGs

Single-cell genomic sequencing of bulk agricultural paddy soil generated 191 million paired-end reads (28.9 Gbp), yielding 384 single-amplified genome (SAG) assemblies. After quality filtering, 187 million high-quality reads (28.1 Gbp) were retained for genome assembly. Initial quality assessment of the complete dataset using CheckM classified the 384 SAGs as 2 high-quality, 124 medium-quality, 212 low-quality, and 46 contaminated SAGs (Supplementary Table S1).

Assembly and quality characteristics for all 384 SAGs are summarized by phylum in Table 1, with per-genome completeness, GC content, genome size, and N50. Patescibacteria was the most frequently recovered phylum (108 SAGs, 28.1% of the dataset), followed by Proteobacteria (55 SAGs), Thermoproteota (33 SAGs), and Acidobacteriota (25 SAGs); together these four phyla accounted for more than half of all recovered genomes. Across the phyla represented by at least nine SAGs, median completeness ranged from 45.0% (Proteobacteria) to 75.9% (Thermoproteota), median GC content from 35.9% (Thermoproteota) to 69.2% (Actinobacteriota), and median genome size from 0.82 Mb (Patescibacteria) to 2.49 Mb (Bacteroidota), with assembly contiguity generally modest throughout (median N50 below 50 kb for most phyla).

**Figure 1:**
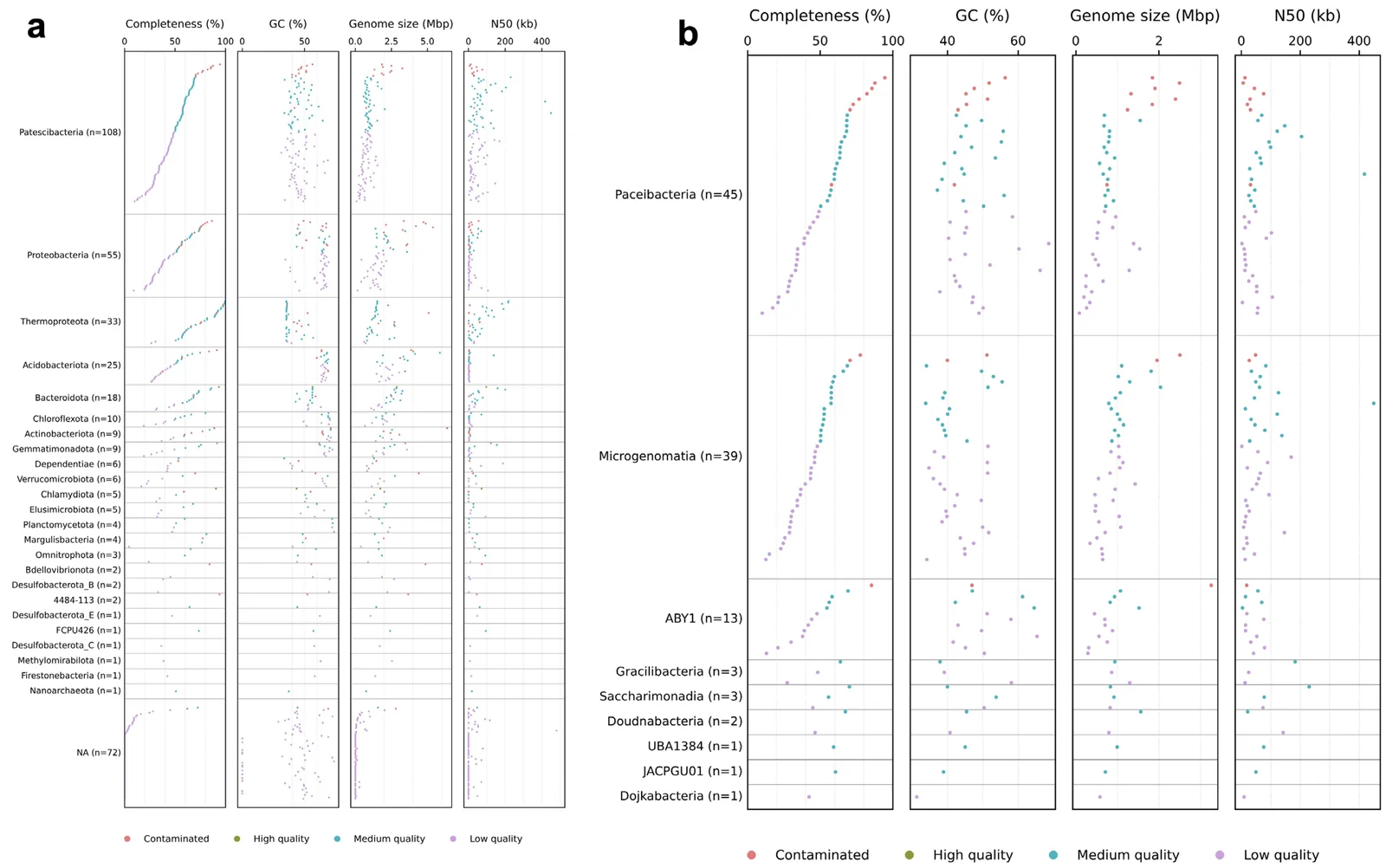
(**a**) Genome-wide assembly and quality characteristics of all 384 SAGs, grouped by phylum. Per-genome completeness, GC content, genome size, and N50 are shown for each of the 384 single-amplified genomes (SAGs) recovered in this study, classified using GTDB-Tk. Genomes are grouped by phylum, with phyla ordered from top to bottom by descending number of recovered SAGs. SAGs for which GTDB-Tk could not assign a phylum-level classification (“NA”) are shown as a separate group at the bottom. Within each phylum, genomes are ordered by completeness (highest to lowest). Points are colored by MIMAG-like quality tier (Contaminated, High quality, Medium quality, Low quality), assigned as defined in Section 2.5, using CheckM completeness and contamination estimates. The number of SAGs recovered for each phylum (*n*) is given alongside its label. (**b**) Class-level composition of the 108 Patescibacteria SAGs, showing the dominance of Paceibacteria and Microgenomatia.

Plotting these values at the level of individual genomes rather than phylum medians (Figure 1a) showed that Patescibacteria’s small genome size was not an artifact of a few incomplete assemblies but a consistent feature of the phylum: SAGs across all quality tiers, from low- to high-quality, clustered toward the smaller end of the genome-size axis, in contrast to Proteobacteria and Acidobacteriota, where larger genomes were recovered even among lower-completeness SAGs. This pattern—small genome size holding regardless of completeness—supports genuine genome streamlining in Patescibacteria rather than assembly fragmentation. The same per-genome view showed Patescibacteria’s GC content and N50 distributions overlapping broadly with those of Proteobacteria and Acidobacteriota, indicating that reduced genome size, rather than lower assembly quality or a distinct nucleotide composition, is what sets this phylum apart in the dataset. Beyond Patescibacteria, the dataset also recovered a substantial number of archaeal SAGs, most notably within Thermoproteota, which included the ammonia-oxidizing genus *Nitrosotalea*—consistent with previous reports [44] linking this lineage to unfertilized paddy soils. As these archaeal lineages fall outside the scope of the present Patescibacteria-focused study, they are not discussed further here.

Class-level analysis of the 108 Patescibacteria SAGs (Figure 1b) showed that the community was dominated by Paceibacteria (*n* = 45) and Microgenomatia (*n* = 39), which together comprised more than three-quarters of the recovered Patescibacteria genomes. ABY1 was the third most frequently recovered class (*n* = 13), while Gracilibacteria (*n* = 3), Saccharimonadia (*n* = 3), Doudnabacteria (*n* = 2), UBA1384 (*n* = 1), JACPGU01 (*n* = 1), and Dojkabacteria (*n* = 1) were each represented by only one to three SAGs. Paceibacteria genomes included the most complete and most contiguous assemblies recovered in this study, reaching up to approximately 90% completeness and N50 values exceeding 400 kb, whereas Microgenomatia genomes showed a slightly higher GC content overall. This class-level distribution indicates that a relatively small number of major Patescibacteria lineages dominate the agricultural paddy soil community despite its overall high phylogenetic diversity.

### 3.2. 16S rRNA Gene Recovery, Phylogenetic Placement, and Pairwise Identity

**Figure 2:**
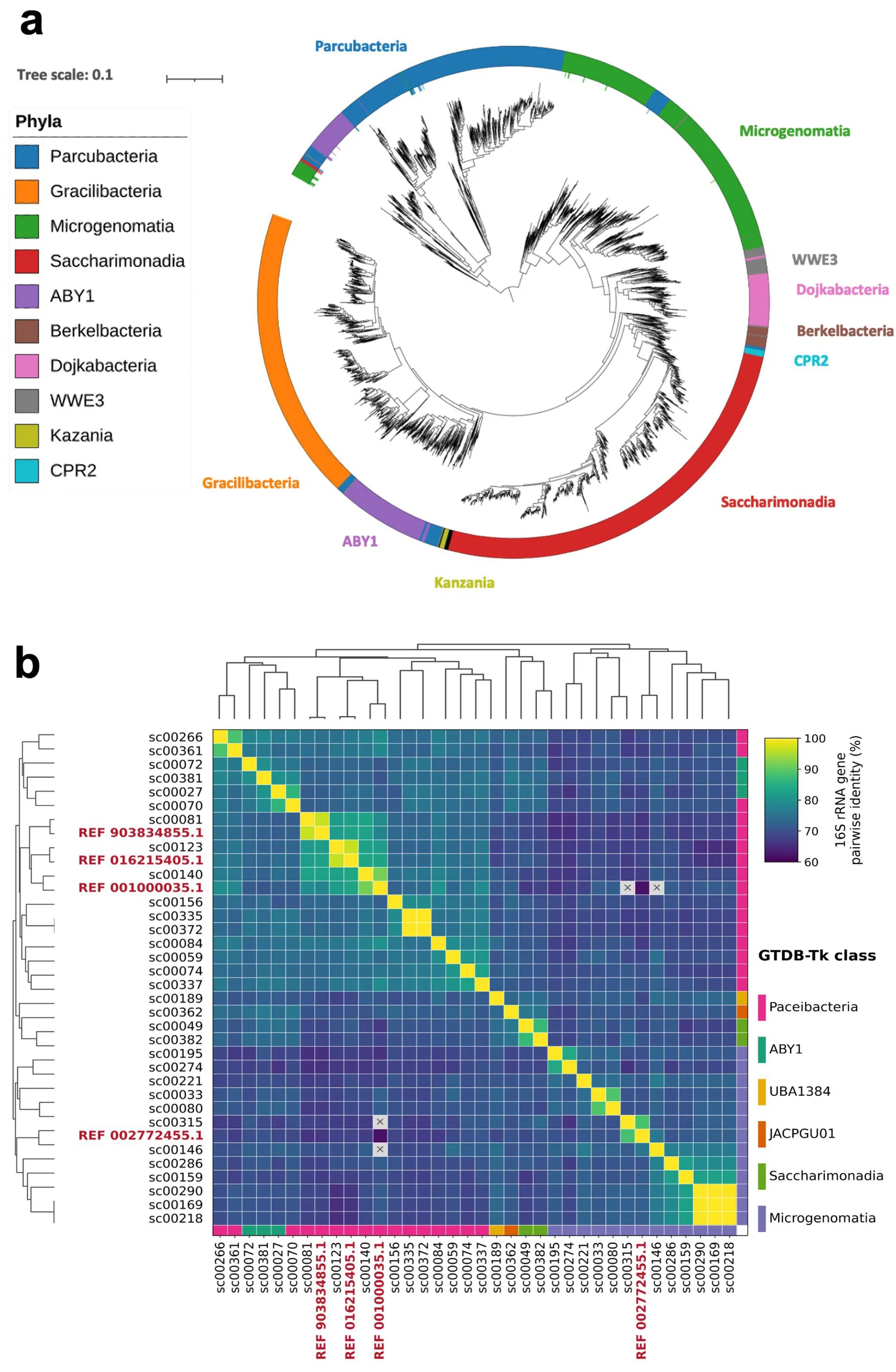
16S rRNA-based diversity and placement of Patescibacteria SAGs. (**a**) Maximum-likelihood phylogenetic tree of 58 near-full-length 16S rRNA gene sequences recovered from the 108 Patescibacteria SAGs, placed within a reference framework comprising 4544 Patescibacteria 16S rRNA gene sequences retrieved from the SILVA 138.2 SSURef NR99 database. Sequences recovered in this study are indicated by ticks on the colored outer stripes, allowing direct comparison of agricultural paddy soil-derived Patescibacteria with the broader phylogenetic diversity represented in SILVA. (**b**) Pairwise 16S rRNA gene identity matrix among the 32 (of 42) focal Patescibacteria SAGs with a detectable 16S rRNA gene and 4 reference genomes (viridis color scale; reference genomes labelled in bold red). Genomes are hierarchically clustered by pairwise identity (average linkage), shown as a dendrogram to the left of the heatmap. The colored strip at the right indicates GTDB-Tk class, listed in the legend in the order classes first appear along the clustered axis. Gray cells marked ‘×’ indicate the two genome pairs for which the overlapping 16S rRNA gene sequence was insufficient to calculate pairwise identity.

To compare genome-resolved taxonomic assignment against ribosomal marker-based phylogenetic assignment, we examined 16S rRNA recovery at two scales. First, across the broader pool of 108 Patescibacteria SAGs (all quality tiers), 58 genomes yielded near-full-length 16S rRNA sequences suitable for phylogenetic placement against the SILVA reference framework (Figure 2a). Of the recovered 16S sequences, Microgenomatia was the most represented class, followed by Parcubacteria (Figure 2a); together these two classes comprised roughly 80% of recovered CPR-affiliated 16S sequences. This ranking differs from the genome-based classification (Figure 1b), where Paceibacteria was the most abundant class and Microgenomatia the second-most abundant—a discrepancy attributable to methodological differences between the two approaches. Genome-based assignment uses all genes present in a SAG, including highly fragmented assemblies lacking rRNA genes, whereas 16S-based analysis is restricted to the subset of SAGs from which ribosomal sequences could be recovered; consequently, 16S-based relative abundance more directly reflects the genomes in which rRNA operons happened to assemble than the overall abundance of the community. Both approaches nonetheless converge on Microgenomatia and Parcubacteria/Paceibacteria as the predominant Patescibacteria lineages in this soil, and their combination offers complementary views of community structure, illustrating both the dominant lineages present and the constraints of ribosomal gene recovery from single-cell assemblies. Importantly, the recovered SAG-derived 16S sequences were distributed across multiple major Patescibacteria lineages within a reference framework of 4544 SILVA sequences, indicating that the recovered genomes represent broad phylogenetic diversity rather than repeated sampling of a few closely related taxa.

For detailed genome-resolved analyses, 42 representative Patescibacteria SAGs were selected based on genome quality and taxonomic representation (Section 2.5). The mean assembled genome size of the 42 selected SAGs was ca. 973 kb, substantially below the 1.4 to 3.2 Mb observed for the remaining dominant bacterial groups (Table 1). Because conventional marker gene-based completeness estimation frequently underestimates genome quality in Patescibacteria genomes, all 42 selected Patescibacteria SAGs were re-evaluated using CheckM2. The reassessment substantially increased genome completeness relative to the original CheckM evaluation from approximately 60% to over 83% and median completeness from approximately 59% to 86% (Supplementary Table S2). Fifteen genomes exceeded 90% completeness following CheckM2 reassessment, whereas none reached this threshold using CheckM. These findings demonstrate that marker gene-based methods substantially underestimate genome completeness in reduced CPR genomes and support the use of CheckM2 estimates throughout subsequent analyses.

As a finer-scale, independent check within the 42-genome focal dataset used for genome-resolved analyses, a 16S rRNA gene was identifiable in only 32 of the 42 SAGs (76%), consistent with the reduced or fragmented rRNA operons already noted for this phylum (Section 3.4). Recovered 16S sequences ranged from 510 to 1592 bp (mean 1346 bp, median 1473.5 bp; a full-length bacterial 16S gene is ~1500 bp). Pairwise sequence identity among the 32 SAGs and four reference genomes (628 of 630 possible pairwise comparisons) averaged 72.1% (median 71.1%; range 62.0 to 100%), indicating extensive phylogenetic diversity within the recovered Patescibacteria (Figure 2b). Only a limited number of genome pairs exceeded commonly accepted genus-level 16S rRNA identity thresholds, while most comparisons fell well below these values. Collectively, these results indicate that the recovered Patescibacteria SAGs represent a taxonomically diverse assemblage composed predominantly of distantly related lineages.

### 3.3. Extensive Amino Acid-Level Divergence Indicates Likely Substantial Taxonomic Novelty

**Figure 3:**
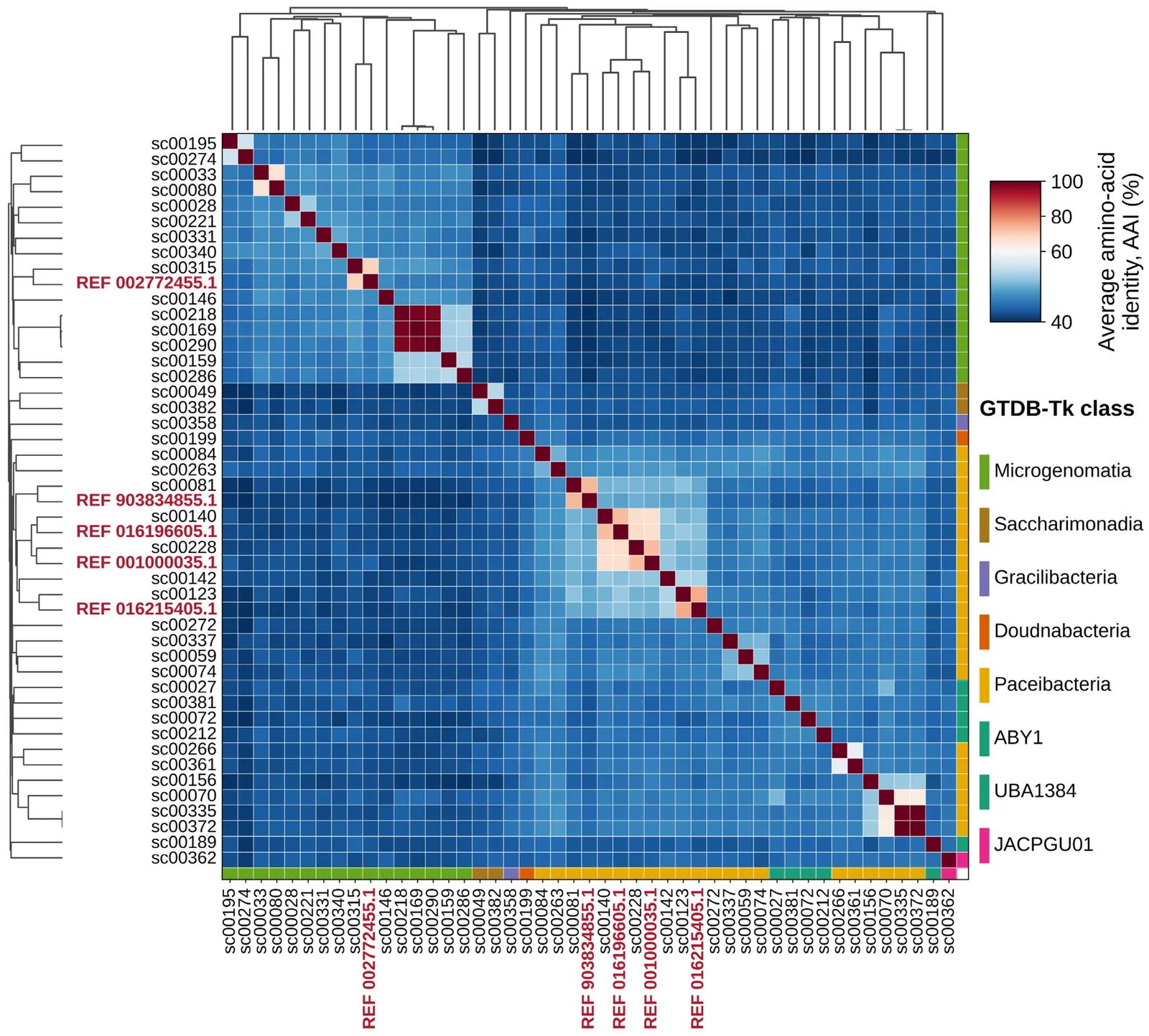
Pairwise average amino acid identity (AAI) matrix among the 42 Patescibacteria SAGs and 5 NCBI reference genomes (47 genomes, 1081 unique pairs), computed using reciprocal-best-hit DIAMOND protein alignments (reference genomes labelled in bold red). Genomes are hierarchically clustered by pairwise AAI (average linkage), shown as dendrograms above and to the left of the heatmap. Cell color indicates pairwise AAI (%), on a diverging blue-white-red scale centered on the permissive 60% genus-level threshold discussed in the text, so that pairs below this threshold appear blue and pairs at or above it appear white-to-red. The colored strip at the right indicates the GTDB-Tk class, listed in the legend in the order classes first appear along the clustered axis. Gray cells indicate genome pairs for which no reciprocal-best-hit protein alignments were identified.

To quantify overall genomic similarity across this population at finer resolution than 16S rRNA alone provides, we computed both ANI and AAI for every pairwise combination of the 42 SAGs and 5 reference genomes. fastANI, which depends on substantial shared nucleotide-level homology, returned a value for only 13 of 2162 ordered genome pairs, reflecting that most pairs in this dataset are too divergent for alignment-based ANI to resolve. AAI, which remains informative at greater evolutionary distances, was successfully computed for all 1081 unique pairs. The resulting AAI distribution had a mean of 44.6% and median of 43.6% (range 40.0–99.9%): only 0.4% of pairs (4/1081) reached the conventional 95% species-level threshold, only 1.5% (16/1081) exceeded a permissive 60% genus-level threshold, and 94.8% of all pairs fell below 50% AAI (Figure 3). Even the five “nearest available” NCBI reference genomes—by definition the closest matches identifiable in current public databases—were only 67–79% identical in AAI to their assigned SAG, well short of any commonly used genus-level threshold. Because no universally agreed-upon AAI or ANI threshold exists for genus- or species-level delineation within Patescibacteria specifically, we present these values descriptively rather than assigning formal taxonomic ranks; nonetheless, the near-complete absence of intermediate-similarity pairs indicates that this single soil sample spans much of the known phylogenetic breadth of the phylum, with minimal redundancy among recovered lineages and substantial likely taxonomic novelty relative to existing reference collections. Thus, Figure 3 demonstrates that the recovered SAG collection is largely composed of distinct evolutionary lineages rather than repeated recovery of closely related genomes, highlighting the exceptional diversity captured from a single soil sample.

This pattern is consistent with, and extends beyond, the 16S-based divergence already noted in Section 3.2.

### 3.4. Phylogenomic Placement and Functional Potential

**Figure 4:**
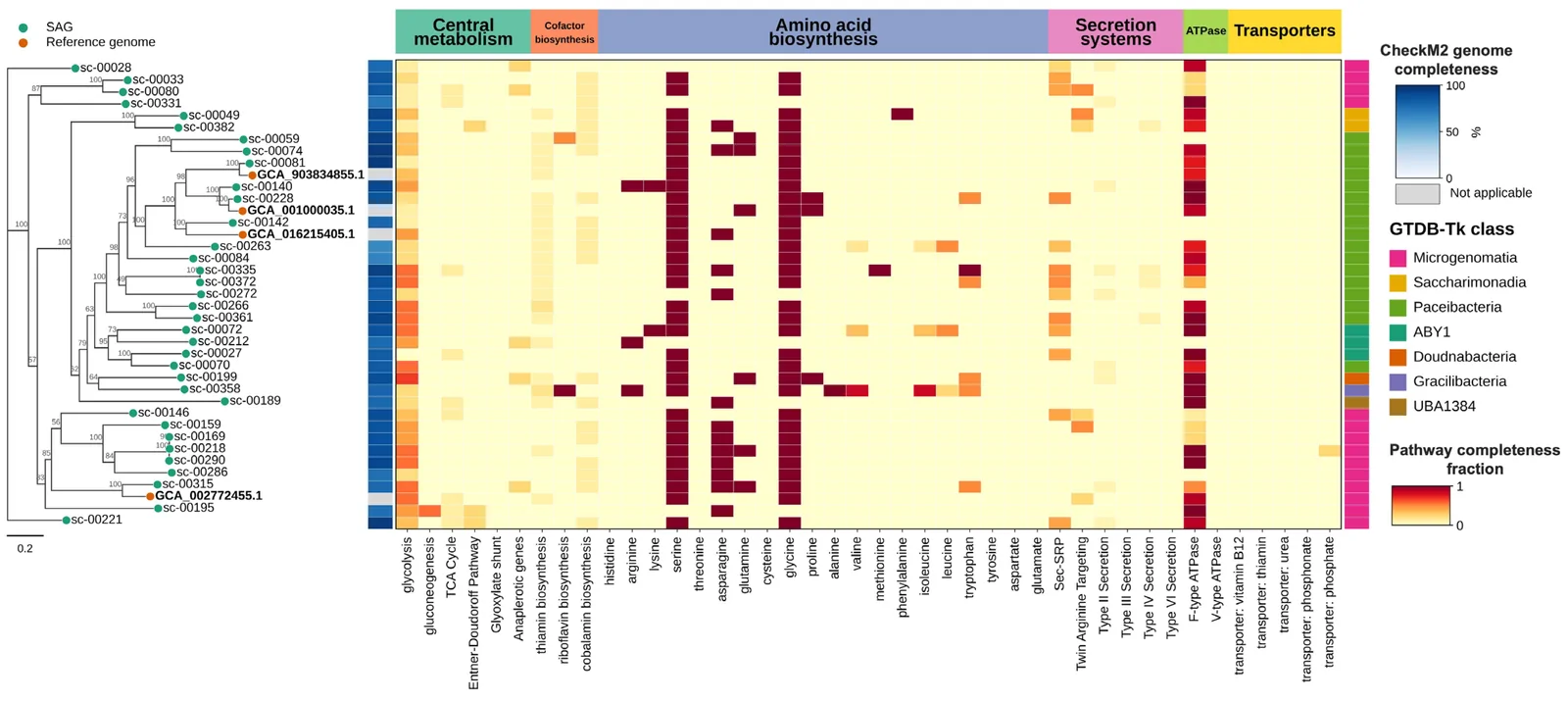
Integrated phylogenomic and metabolic reconstruction of Patescibacteria SAGs. Maximum likelihood phylogeny based on concatenated rp16 ribosomal proteins for 35 Patescibacteria SAGs (green tip circles) and four NCBI reference genomes (orange tip circles, bold labels), with ultrafast bootstrap support (1000 replicates) shown at internal nodes. The colored strip immediately to the right of the tree shows CheckM2 genome completeness for each genome (grey = not applicable, reference genomes). KEGG-Decoder pathway/module completeness (fraction, 0–1) is presented as an aligned heatmap for 42 pathways/modules, grouped into six functional categories, indicated by the colored, labelled header above the heatmap; the colored strip at the right of the heatmap indicates GTDB-Tk class.

Standard multi-marker phylogenomic schemes built around roughly 120 largely ribosomal bacterial marker genes are, for the same reasons noted in Section 3.1, unreliable for lineages with reduced ribosomal gene complements. We therefore constructed a phylogeny from the 16-marker rp16 set. Thirty-five of the 42 Patescibacteria SAGs retained at least 8 of the 16 markers and were included in the tree, together with four of the five retrieved NCBI reference genomes, yielding a final maximum-likelihood tree of 39 taxa (concatenated alignment length 2040 amino acids; 1000 ultrafast bootstrap replicates). In every case, the reference genome fell phylogenetically adjacent to the SAG(s) to which it had previously been assigned, supporting both the original reference assignment and the rp16-based tree topology, consistent with the AAI- and 16S-based placements above (Section 3.2 and Section 3.3). Completeness values annotated at each tree tip are CheckM2 estimates (Section 3.1), which for this same set of 35 SAGs average 87.0% (range 63.7–100%)—substantially higher than the corresponding CheckM1 values (mean 60.9%, range 50.2–70.1%)—and are shown alongside a KEGG pathway/module completeness heatmap for the same genomes (Figure 4). Integration of the phylogeny with KEGG pathway completeness revealed broadly conserved but reduced metabolic repertoires across the recovered genomes, while lineage-specific variation in pathway completeness indicated functional differentiation among major phylogenetic groups.

### 3.5. A Conserved, Translation-Focused, Biosynthetically Streamlined Gene Repertoire Spans This Phylogenetic Diversity

**Figure 5:**
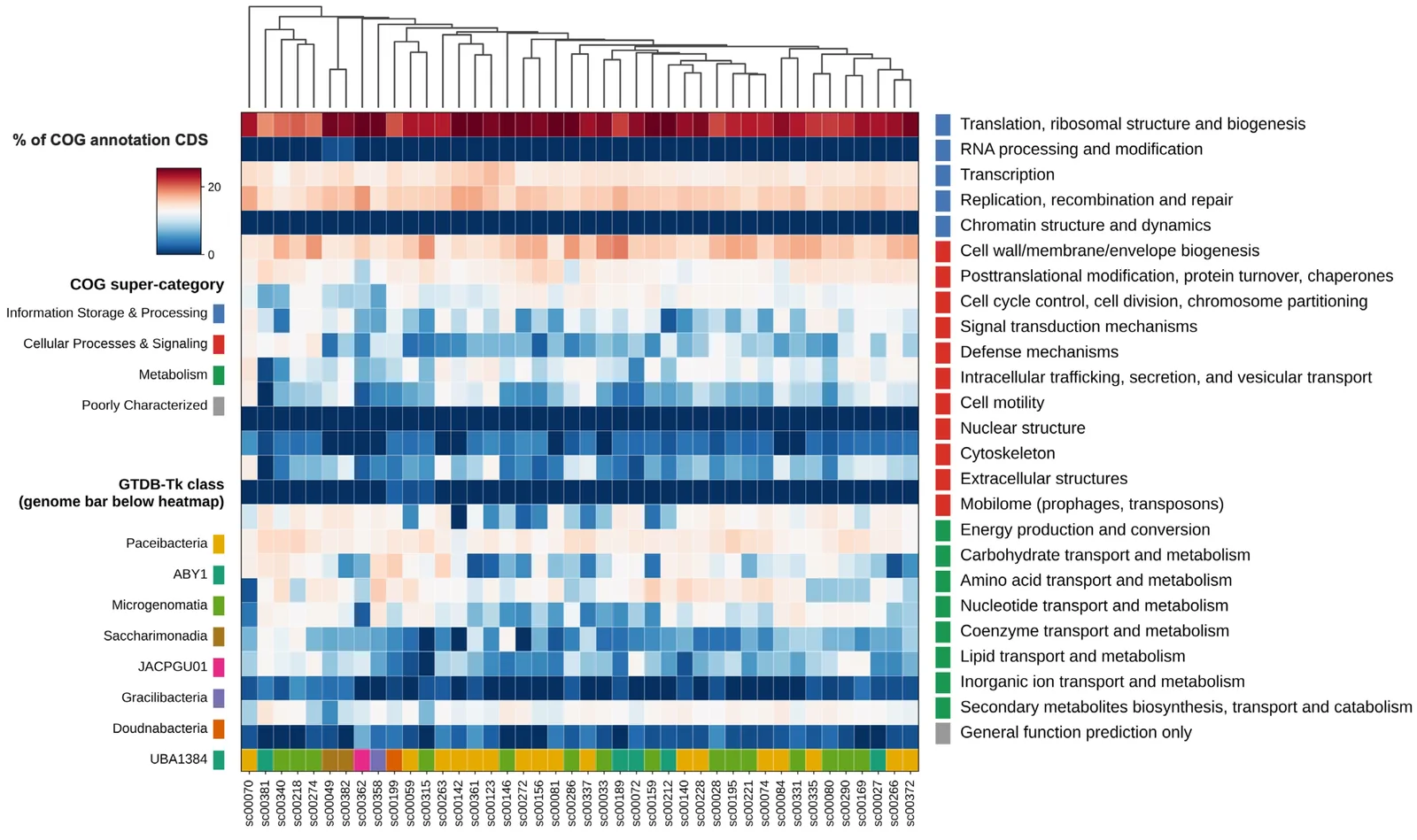
Per-genome COG functional category heatmap for the 42 Patescibacteria SAGs. Genomes (columns) are ordered by hierarchical clustering (average linkage, Euclidean distance on z-scored category profiles) of their COG-category profiles, shown as a dendrogram above the heatmap; genome IDs and GTDB-Tk class (colored strip) are shown below the heatmap. COG categories (rows) are grouped by COG super-category indicated by the colored strip and labels to the right of the heatmap. Cell color indicates the percentage of COG-annotated coding sequences assigned to each category, on a diverging blue-white-red scale. The legend at left gives COG super-category and GTDB-Tk class colors, the latter listed in the order classes first appear along the clustered genome axis.

Despite this extensive phylogenetic and genomic divergence, the functional composition of the 42 genomes was remarkably uniform. COG category assignments (Figure 5) showed that translation, ribosomal structure and biogenesis (category J) accounted for approximately 28% of all COG-annotated coding sequences, far exceeding any other functional category; the next most represented categories were cell wall/membrane/envelope biogenesis (M, ~10–12%), replication, recombination and repair (L, ~10%), and transcription (K), carbohydrate transport and metabolism (G), and posttranslational modification/protein turnover/chaperones (O), each at roughly 6–8%. Categories directly associated with core biosynthetic metabolism—energy production (C), amino acid transport and metabolism (E), nucleotide transport and metabolism (F), coenzyme transport and metabolism (H), inorganic ion transport and metabolism (P), and secondary metabolite biosynthesis (Q)—were each under 5% and mostly under 2%.

KEGG ortholog assignment and pathway/module completeness scoring reinforced this picture in more granular metabolic terms. Averaged across the 42 SAGs, several core amino acid biosynthesis pathways were essentially undetectable (mean completeness fraction 0.00 for histidine, threonine, cysteine, tyrosine, aspartate, and glutamate biosynthesis), and most others were only marginally represented (0.04–0.10 for arginine, lysine, proline, methionine, phenylalanine, isoleucine, leucine, valine, and tryptophan biosynthesis; 0.17–0.31 for glutamine and asparagine). The tricarboxylic acid cycle, cofactor biosynthesis pathways (thiamin, riboflavin, and cobalamin biosynthesis each averaged only 0.05–0.06), and bacterial secretion systems (Type II through Type VI) were likewise nearly absent across the population. In marked contrast, serine and glycine biosynthesis were broadly retained (mean completeness fraction 0.81 for each), as was the F-type ATP synthase, present at meaningful completeness in most genomes. This translation-dominated, biosynthetically minimal gene repertoire, with retention of serine/glycine metabolism and core energy-conserving ATP synthase machinery, is consistent with the established model of Patescibacteria as metabolically streamlined organisms likely dependent on host cells or co-occurring community members for many essential biosynthetic products [2,8], and extends this generalization to an agricultural paddy-soil system at single-cell resolution—consistent with a broader observation that CPR/Patescibacteria genomes recovered from very different soils (Amazonian tropical forest and pasture soils, permafrost active-layer soils) share a similar overall functional profile distinct from co-occurring, non-CPR soil bacteria [45].

### 3.6. Orthology-Based Comparative Genomics Reveals a Minimal Shared Core Beyond COG/KEGG Annotation

**Figure 6:**
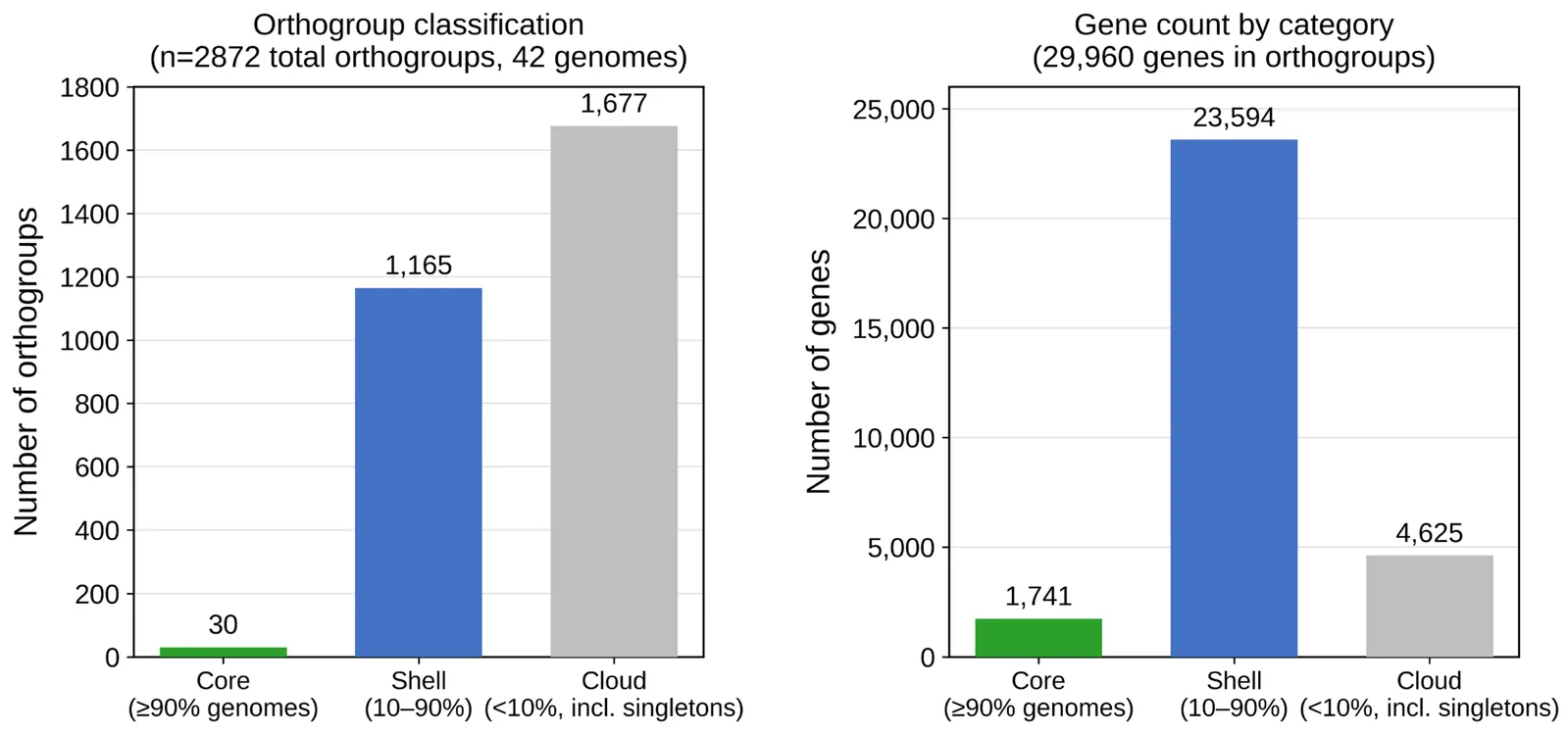
Orthogroup classification for the 42 Patescibacteria SAGs (OrthoFinder), shown as (**left**) the number of orthogroups and (**right**) the number of genes falling into core (≥90% of genomes), shell (10–90%), and cloud (<10%, including singletons) categories.

Because COG and KEGG assignments cover only a subset of predicted proteins, we additionally clustered all predicted proteins from the 42 SAGs into orthogroups with OrthoFinder, independent of any functional database. This assigned 29,960 of 41,724 predicted proteins (71.8%) to 2872 orthogroups; the remainder were unclustered, genome-specific genes. Classifying orthogroups by the fraction of genomes in which they occurred showed a small core (30 orthogroups, 1.0% of all orthogroups, present in ≥90% of genomes; 1741 genes, 5.8% of clustered genes), a much larger shell of intermediate-occurrence orthogroups (1165 orthogroups present in 10–90% of genomes; 23,594 genes, 78.8%), and a substantial cloud of rare or genome-specific orthogroups (1677 orthogroups present in <10% of genomes, including singletons; 4625 genes, 15.4%; Figure 6). Notably, no single orthogroup was detected in all 42 genomes (the single most widely shared orthogroup occurred in 41 of 42), reinforcing at the gene-family level the same pattern of minimal redundancy seen in the AAI and 16S comparisons above; because most SAGs are not fully complete even after CheckM2 reassessment, we defined “core” using a ≥90% occurrence threshold rather than requiring 100% presence, to reduce sensitivity to residual incompleteness in a subset of genomes.

Cross-referencing the 70 most broadly shared orthogroups (each detected in at least 30 of the 42 genomes) against existing product and COG annotations (Supplementary Figure S1) showed that the large majority carried an interpretable functional label rather than being annotated only as “hypothetical protein”: 9 orthogroups corresponded to individual ribosomal proteins, a further 12 to other translation- or RNA-processing-related genes, 15 to DNA replication/repair and recombination genes (e.g., RecA, RecG, RuvC, the UvrABC excision repair system, DNA topoisomerase I), 5 to cell-division- or cell-wall-associated genes (e.g., MreB, the peptidoglycan transpeptidase MrdA), 6 to secretion- or membrane-transport-associated genes (e.g., SecY, YidC, prepilin peptidase), 4 to transcription-associated genes, and 2 to chaperone/protein-quality-control functions; only 15 of the 70 (21%) remained dominated by unannotated “hypothetical protein” calls. This orthology-based view is independent of, but fully consistent with, the COG category-level result in Section 3.5: it confirms at the level of individual, cross-genome-validated gene families that the translation apparatus is the most conserved functional module in this population, while additionally highlighting a comparably well-conserved DNA replication/repair and genome-maintenance module not separately called out by the coarser COG category summary.

### 3.7. Recovery of Virus-Dominated Genomes

In principle, this single-cell sorting approach can recover non-cellular genomes in addition to bacterial and archaeal SAGs. geNomad analysis for all SAGs identified four SAGs whose assemblies were predominantly composed of virus-classified contigs (Table 2). Functional annotation indicated that these assemblies were most closely related to members of the Nucleocytoviricota (giant viruses), while the majority of virus-associated sequences detected in other SAGs represented only minor prophage fragments. The detailed analyses are provided in the Supplementary Table S3.

### 3.8. Analysis of Contaminated Patescibacteria SAGs

Notably, 11 of the 46 contaminated SAGs were assigned to Patescibacteria. Because Patescibacteria genomes are often characterized by a parasitic lifestyle and limited metabolic capacities, we examined these 11 SAGs separately to determine whether elevated CheckM contamination reflected DNA originating from multiple organisms rather than their host genome DNA (Table 3). Ten of the 11 SAGs contained at least one detectable 16S rRNA gene, and five contained two or more 16S rRNA gene copies. Within each multi-copy SAG, the co-occurring 16S rRNA sequences were highly divergent, with pairwise identities substantially below the level expected for intragenomic rRNA operon copies. Independent taxonomic classification further identified 16S rRNA sequences assigned to non-Patescibacteria lineages in several SAGs, supporting the co-assembly of DNA from taxonomically distinct organisms. Whether these co-encapsulated bacteria represent genuine hosts or symbiotic partners of Patescibacteria, or were instead incidentally co-sorted into the same droplet, cannot be resolved from these data alone. Notably, several of the divergent 16S rRNA sequences recovered from these SAGs were themselves affiliated with Patescibacteria (albeit distinct sub-lineages), consistent with the incidental co-recovery of more than one Patescibacteria cell in some cases; in others, the contaminating sequences were affiliated with unrelated phyla, including Burkholderiales-associated Proteobacteria, leaving open the possibility of a genuine physical association in at least some instances. The full results are provided in Supplementary Table S4.

## 4. Discussion

### 4.1. Agricultural Paddy Soil as a Habitat for Patescibacteria

The present study generated a genome-resolved view of the microbial diversity present in agricultural paddy soil through single-cell genomics. The 384 SAGs recovered from a single soil sample represented a broad range of bacterial and archaeal lineages, spanning 24 phyla and numerous uncultivated taxonomic groups. Notably, 69 SAGs were singletons at the genus level in the dataset, indicating that a substantial fraction of the recovered genomes represented distinct genus-level lineages. This high degree of taxonomic heterogeneity underscores the breadth of microbial genomic diversity captured by single-cell sequencing and provides genomic access to a wide range of poorly characterized microorganisms from a complex soil environment. Within this diverse collection, several taxonomic patterns were particularly notable, including the frequent recovery of *Nitrosotalea*, the repeated recovery of the uncultivated UBA10030 lineage, and the substantial representation of Patescibacteria.

Agricultural soil, and paddy soil in particular, is a distinctive ecological setting for Patescibacteria relative to groundwater, engineered systems, and host-associated environments. It is characterized by extreme spatial heterogeneity at the micrometer scale, steep nutrient gradients, dynamic moisture regimes—compounded, in a paddy system, by a recurring flood–drain cycle—and well-structured microbial communities. These characteristics create localized microhabitats in which metabolically simplified organisms can persist in close association with other microbes, even without extensive biosynthetic capacity, as reported by Nunan [46] and Santana-Pereira et al. [47]. This heterogeneity may also help explain the unexpectedly high representation of Patescibacteria in this single sample. Sampling took place shortly after rice harvest, once the field had been drained and the plant-derived carbon and nutrient inputs that support many other soil heterotrophs were removed; this transition may have transiently favored metabolically streamlined, scavenging organisms such as Patescibacteria over more self-sufficient competitors. However, without a comparable sample from a different point in the flood–drain cycle or from a still-flooded, actively cropped plot, we cannot distinguish this possibility from a more general or stable feature of this soil’s microbial community.

The dominance of Paceibacteria and Microgenomatia among genome-resolved SAGs, and of Microgenomatia and Parcubacteria among 16S-recovered sequences, suggests that these lineages are especially well adapted to heterogeneous, dynamic soil environments. Both are repeatedly recovered in terrestrial and subsurface systems [7,15] and are thought to rely on adaptive metabolic exchange rather than obligate host selectivity—a strategy that may be advantageous where diverse bacterial hosts are in close physical proximity on mineral surfaces and within soil aggregates. In contrast, Saccharimonadia, which is commonly prevalent in host-rich or high-cell-density habitats, was only a minor constituent of this dataset, suggesting that agricultural/paddy soils may limit the abundance of CPR taxa with more specific host partnerships or that depend on high microbial cell density [48,49]. The consistently low representation of Berkelbacteria and Dojkabacteria [2,49] further suggests that these lineages occupy narrower ecological niches. Collectively, these observations indicate that the Patescibacteria community recovered from this paddy soil is dominated by a limited number of lineages, primarily Paceibacteria, Microgenomatia, and Parcubacteria, whereas other Patescibacteria groups were comparatively rare.

### 4.2. Comparative Insights Across Environments

Patescibacteria have repeatedly been reported at high relative abundance in subsurface and groundwater environments [14,50,51], providing an important point of reference for the patterns observed here. Genome-resolved groundwater studies have reported Patescibacteria fractions as high as ~31% of the community at the aquifer scale, and up to 79% locally within individual wells [14]. Notably, Herrmann et al. [14] documented niche partitioning within the phylum, with Parcubacteria more abundant in groundwater-fed sand filters and Microgenomatia more common in aquifer sediments; the strong representation of both lineages in this agricultural paddy soil dataset suggests they are not confined to chemically stable subsurface habitats but are also well adapted to terrestrial settings with greater spatial heterogeneity and fluctuating resource availability.

By contrast, the comparatively low abundance of Saccharimonadia in this dataset stands in contrast to its prominence in high-cell-density, host-rich environments: activated-sludge studies have shown that Saccharimonadia and Parcubacteria together account for up to 80% of CPR-affiliated reads in wastewater treatment plants [9], and microscopy has shown that the Saccharimonadia representative TM7x interacts epibiotically with *Actinomyces* in the human oral cavity, underscoring a dependence on intimate host relationships [52]. Berkelbacteria and WS6 (Dojkabacteria) were likewise recovered only at very low abundance here, mirroring their minor representation even in highly CPR-enriched engineered systems—for example, size-fractionated analyses of activated sludge found Berkelbacteria and Dojkabacteria to remain minor constituents even where total Patescibacteria reached 73.5% of the community [49]. These consistently low abundances across natural and engineered systems suggest that such taxa occupy narrow ecological niches with strict host or substrate selectivity that constrains their proliferation even under otherwise CPR-favorable conditions.

A further point of comparison concerns functional gene content specifically. Santana-Pereira et al. [47] reconstructed 27 CPR MAGs from the Cullars Rotation, a long-term agricultural soil-fertility experiment in Alabama, USA, and reported that CPR community composition was not strongly restructured by soil nutrient regime except under extreme conditions—offering some reassurance that a single sample from an unfertilized plot is not necessarily an unrepresentative snapshot with respect to fertilization status specifically. However, their CPR genomes were enriched in enzymes predicted to degrade plant-derived carbohydrates and included possible aerotolerance- or aerobic-respiration-associated genes. Our KEGG-Decoder screen found essentially none of these features: carbohydrate-active enzymes such as cellulase, chitinase, and pectinesterase were undetected across all 42 SAGs (with only rare, partial hits for glucoamylase and generic starch/glycogen degradation), complete aerobic respiratory-chain components (cytochrome oxidases) were essentially absent, and no hydrogenase of any type was detected in any genome. We cannot determine from the present data alone whether this contrast reflects a genuine ecological or physiological difference—our sample was collected after rice harvest with the paddy water already drained, capturing a community transitioning from a flooded, anoxic growing-season regime toward a more oxic state, which may select for different accessory metabolic capacities than a continuously managed, non-paddy agricultural soil—or whether it instead reflects residual incompleteness of SAGs even after CheckM2 reassessment, which could disproportionately miss lower-copy accessory genes relative to the core genes captured in COG/KEGG summary statistics. We present this comparison as an open question rather than a conclusion.

Collectively, the repeated prevalence of Parcubacteria/Paceibacteria and Microgenomatia across groundwater, subsurface, and now agricultural paddy soil settings points to substantial ecological versatility and wide environmental adaptability for these lineages. In contrast, the lower abundance of Saccharimonadia and several other CPR lineages may reflect a greater dependence on specific microbial hosts rather than environmental nutrient availability. Whether the phylogenetic breadth of Patescibacteria recovered here reflects a genuine ecological peculiarity of agricultural paddy soil—for example, the fine-scale, flood–drain-cycled microhabitat heterogeneity discussed above—or is instead a general feature of Patescibacteria diversity that would be recovered from comparably intensive single-cell sampling of almost any soil, cannot be resolved from a single sample and remains an open question for future comparative studies.

### 4.3. Methodological Implications: CheckM Versus CheckM2 for CPR Genome-Quality Assessment

Genome completeness estimates obtained with CheckM2 substantially improved the interpretation of the recovered Patescibacteria SAGs by indicating that many genomes were considerably more complete than suggested by conventional marker gene-based assessment. This reassessment increased confidence in the subsequent phylogenomic and functional analyses and supports the inclusion of these genomes in comparative studies of CPR bacteria. As CheckM2 has become the standard approach for assessing genome quality in reduced microbial genomes, our findings are consistent with recent studies applying composition-based completeness estimation to CPR lineages. Notably, none of the 42 focal SAGs met the >90% completeness threshold under CheckM alone, whereas 15 did so under CheckM2. Had we relied on CheckM alone, our phylogenomic and functional-annotation analyses would have been restricted to a set of genomes, none of which would have qualified as near-complete, materially understating the true genome quality and phylogenetic coverage achieved in this study.

### 4.4. A Conserved Reduced-Genome Lifestyle Amid Extensive Phylogenetic Novelty

Despite the extensive phylogenetic diversity recovered from a single agricultural paddy soil sample, the genomes shared a remarkably conserved functional profile characterized by reduced biosynthetic capacity and a translation-focused gene repertoire. This is consistent with genome reduction being an ancient and conserved feature of the radiation rather than a lineage-specific adaptation to this particular environment, and it extends, to a flooded/drained paddy-soil system at single-cell resolution, the broader pattern of functional convergence previously documented for CPR genomes recovered from otherwise very different soils [45].

### 4.5. Recovery of Virus-Dominated SAGs

The finding of a small number of apparent giant-virus genomes demonstrates that unbiased single-cell approaches can simultaneously provide insights into both cellular and viral components of complex soil microbial communities (Table 2). Because only four virus-dominated SAGs were identified, detailed characterization is presented in the Supplementary Materials.

### 4.6. Limitations and Future Directions

This study has several limitations that constrain the strength of any inference. It is based on a single soil sample—one experimental plot, one 7–8 cm depth horizon, and one collection date, shortly after rice harvest with the paddy water already drained. No paired sample from a fertilized plot, nor any additional bulk-soil timepoint spanning the flood–drain cycle, was available for direct comparison within the scope of this study. Multiple-displacement whole-genome amplification is also known to introduce coverage bias and chimera formation, which can locally inflate or deflate apparent gene copy number and contribute to the assembly fragmentation seen across these SAGs; while our CheckM2-based completeness reassessment and contamination screening (Section 2.14) mitigate some of these effects, we cannot fully exclude a residual influence of amplification bias on genome completeness and functional-repertoire estimates. As with any SAG-based analysis, the apparent absence of a gene or pathway can never be fully distinguished from incomplete genome recovery, even for genomes reassessed as near-complete by CheckM2; conclusions about the “absence” of specific functions (for example, hydrogenases) should accordingly be read as provisional. Similarly, a 16S rRNA gene could be identified in only 32 of the 42 focal SAGs, and recovered sequences varied substantially in length (510–1592 bp); this incomplete and uneven recovery limits the resolution and confidence of 16S-based phylogenetic placement relative to the rp16 marker-based phylogenomic tree, and pairwise identity comparisons involving the shortest sequences should be interpreted cautiously. Finally, because AAI and ANI reference thresholds for genus- and species-level delineation are not yet standardized for Patescibacteria, our diversity statistics are best interpreted descriptively rather than as formal counts of novel genera or species.

Future work that would help resolve the open questions raised here includes sampling additional timepoints across the paddy flood–drain cycle and, ideally, a matched fertilized plot, to test whether the carbohydrate-degradation and respiratory gene-content contrast with Santana-Pereira et al. [47] reflects redox history or fertilization regime; deeper or longer-read sequencing of individual SAGs to test whether the apparent absence of hydrogenases and complete respiratory chains persists in more complete assemblies; and metatranscriptomic or metaproteomic data from the same soil to test whether the retained core (translation, serine/glycine metabolism, F-type ATP synthase) is also the actively expressed core in situ. More broadly, extending single-cell genomics of Patescibacteria to metagenomic, transcriptomic, and experimental validation approaches would help clarify the metabolic interactions and ecological functions of this phylum in agricultural soil and rhizosphere systems.

## 5. Conclusions

This study presents a genome-resolved, single-cell genomic analysis of Patescibacteria in agricultural paddy soil. An unbiased, non-selective single-cell survey of bulk soil from an unfertilized, recently drained paddy plot recovered Patescibacteria as the single most abundant phylum among reliable genomes (42 of 126, and 108 of 384 across all quality tiers), with genome sizes far smaller than any co-occurring lineage. Class-level analysis showed clear dominance by Paceibacteria and Microgenomatia, with other CPR lineages consistently in low abundance—a pattern broadly consistent with, yet also distinct from, previously characterized CPR communities in groundwater, subsurface, and engineered systems. Reassessment with CheckM2 showed that these genomes are substantially more complete than standard CheckM estimates suggest, resolving an apparent quality problem that was in fact a tool artifact and reinforcing a broader methodological caution for CPR genome-quality assessment. Phylogenomic and genome-similarity analyses showed that this single sample spans much of the known diversity of the phylum, with minimal redundancy among recovered lineages and substantial likely taxonomic novelty relative to public reference genomes. Despite this diversity, gene content was highly conserved and consistent with the established model of Patescibacteria as translation-focused, biosynthetically dependent organisms, extending this generalization to an agricultural paddy-soil system. Together, these findings illustrate both a practical methodological lesson for genome-quality assessment of CPR lineages and a substantive contribution to the still-limited characterization of Patescibacteria diversity and gene content in agricultural soils, while establishing the value of single-cell genomics for revealing latent CPR diversity in terrestrial, actively managed soil ecosystems.

## Figures and Tables

**Table 1:** Genome-wide assembly and quality characteristics of all 384 SAGs by phylum, based on GTDB-Tk classification. H/M/L/C = number of SAGs classified as High/Medium/Low-quality or Contaminated under the MIMAG-like criteria used in Section 2.5. Completeness and contamination were estimated with CheckM; genome size, GC content, and N50 were computed with QUAST. Phyla are ordered by descending number of recovered SAGs (*n*). The “Unclassified” row corresponds to SAGs for which GTDB-Tk could not assign a phylum-level classification.

Phylum	*n*	Quality (H/M/L/C)	Completeness (%) Median (Range)	GC (%) Median (Range)	Genome Size (Mb) Median (Range)	N50 (kb) Median (Range)
Patescibacteria	108	0/42/55/11	48.7 (10.0–94.5)	45.0 (31.3–68.7)	0.82 (0.08–3.26)	44 (2–449)
Proteobacteria	55	0/13/30/12	45.0 (9.1–86.4)	64.5 (34.7–69.9)	1.75 (0.15–5.38)	5 (1–106)
Thermoproteota	33	0/25/2/6	75.9 (26.5–99.5)	35.9 (35.3–56.8)	1.45 (0.61–5.09)	37 (1–217)
Acidobacteriota	25	0/8/13/4	45.5 (26.7–91.4)	65.9 (59.8–69.0)	2.40 (1.48–5.87)	3 (1–138)
Bacteroidota	18	1/11/5/1	67.2 (31.0–93.4)	55.2 (42.9–63.0)	2.49 (1.11–3.27)	36 (3–200)
Chloroflexota	10	0/5/5/0	49.8 (19.0–80.1)	69.0 (49.2–70.3)	1.90 (0.65–2.69)	5 (1–70)
Actinobacteriota	9	0/2/3/4	59.5 (36.7–88.8)	69.2 (64.2–71.1)	2.44 (1.41–6.37)	4 (2–10)
Gemmatimonadota	9	0/3/5/1	49.3 (19.1–91.6)	66.1 (61.6–72.3)	1.99 (1.21–3.58)	6 (1–155)
Dependentiae	6	0/1/4/1	44.1 (42.1–53.5)	40.0 (33.6–48.1)	1.06 (0.80–1.46)	15 (6–188)
Verrucomicrobiota	6	0/1/4/1	30.5 (16.6–69.8)	65.3 (58.8–68.5)	1.85 (1.13–4.40)	14 (1–42)
Chlamydiota	5	1/1/2/1	51.0 (31.2–90.5)	50.6 (43.6–54.8)	1.31 (0.75–2.01)	1 (0–72)
Elusimicrobiota	5	0/2/3/0	36.4 (32.2–67.8)	58.2 (46.8–65.8)	1.06 (0.73–2.12)	27 (10–93)
Planctomycetota	4	0/2/2/0	49.9 (47.2–59.5)	72.3 (72.1–73.7)	2.07 (1.64–2.39)	4 (3–5)
Margulisbacteria	4	0/2/1/1	77.0 (4.4–81.0)	48.5 (40.4–50.9)	1.48 (0.38–1.97)	34 (22–46)
Omnitrophota	3	0/2/1/0	59.7 (23.9–65.3)	44.6 (43.9–59.1)	1.62 (0.88–1.84)	59 (11–92)
Bdellovibrionota	2	0/0/1/1	64.8 (45.4–84.2)	56.1 (55.5–56.6)	3.35 (1.85–4.86)	38 (3–73)
Desulfobacterota_B	2	0/0/2/0	35.7 (33.2–38.1)	69.8 (69.4–70.1)	2.46 (2.22–2.69)	5 (1–8)
4484-113	2	0/1/0/1	79.2 (64.3–94.0)	48.2 (44.2–52.3)	2.56 (1.45–3.66)	53 (46–61)
Desulfobacterota_E	1	0/0/1/0	47.1 (47.1–47.1)	62.2 (62.2–62.2)	1.05 (1.05–1.05)	48 (48–48)
FCPU426	1	0/1/0/0	73.6 (73.6–73.6)	57.1 (57.1–57.1)	2.41 (2.41–2.41)	95 (95–95)
Desulfobacterota_C	1	0/0/1/0	36.4 (36.4–36.4)	57.9 (57.9–57.9)	1.69 (1.69–1.69)	10 (10–10)
Methylomirabilota	1	0/0/1/0	38.5 (38.5–38.5)	62.9 (62.9–62.9)	2.54 (2.54–2.54)	8 (8–8)
Firestonebacteria	1	0/0/1/0	42.3 (42.3–42.3)	58.2 (58.2–58.2)	1.38 (1.38–1.38)	15 (15–15)
Nanoarchaeota	1	0/1/0/0	50.9 (50.9–50.9)	37.4 (37.4–37.4)	0.75 (0.75–0.75)	19 (19–19)
Unclassified	72	0/1/70/1	0.0 (0.0–72.5)	45.4 (0.0–73.6)	0.00 (0.00–2.80)	0 (0–480)

**Table 2:** Summary of four SAGs with predominantly virus-classified assemblies identified by geNomad.

Single Amplified Genomes (SAGs)	Viral Fraction of Assembly	Viral Length/Total Length	*n*_hallmarks	Max virus_score	CheckM Completeness	GTDB-Tk Taxonomy
220926_sc-00278	63.3%	426,197/673,451 bp	7	0.978	3.38%	Unclassified Bacteria
220926_sc-00129	61.3%	480,451/784,103 bp	10	0.953	2.73%	Unclassified Archaea
220926_sc-00098	57.8%	41,743/72,279 bp	0	0.979	0.00%	No classification (geNomad: Mimiviridae)
220926_sc-00209	57.2%	335,542/586,273 bp	1	0.980	8.38%	Unclassified Archaea

**Table 3:** Comparison of contaminant-derived 16S rRNA taxonomy and host GTDB-Tk taxonomy in CheckM-contaminated single-cell genomes.

genome_id	Completeness (%)	Contamination (%)	Detected 16S	gtdbk_taxonomy	Confidence
220926_sc-00031	82.18	37.41	1	d__Bacteria;p__Patescibacteria;c__Paceibacteria;o__UBA6257;f__2-01-FULL-56-20;g__MHLB01;s__	
			2	d__Bacteria;p__Patescibacteria;c__Parcubacteria;o__Candidatus_Jorgensenbacteria;f__Candidatus_Jorgensenbacteria;g__Candidatus_Jorgensenbacteria;s__	0.9976
220926_sc-00035	77.58	42.73	1	d__Bacteria;p__Patescibacteria;c__Microgenomatia;o__2-02-FULL-39-11;f__2-02-FULL-39-11;g__;s__	
			2	d__Bacteria;p__Patescibacteria;c__Microgenomatia;o__Candidatus_Gottesmanbacteria;f__Candidatus_Gottesmanbacteria;g__Candidatus_Gottesmanbacteria;s__	1
220926_sc-00036	70.53	60.74	1	d__Bacteria;p__Patescibacteria;c__Microgenomatia;o__GWA2-44-7;f__UBA8517;g__;s__	
			2	d__Bacteria;p__Patescibacteria;c__Microgenomatia;o__Candidatus_Woesebacteria;f__Candidatus_Woesebacteria;g__Candidatus_Woesebacteria;s__	1
			3	d__Bacteria;p__Patescibacteria;c__Microgenomatia;o__Candidatus_Woesebacteria;f__Candidatus_Woesebacteria;g__Candidatus_Woesebacteria;s__	1
220926_sc-00134	72.69	18.93	1	d__Bacteria;p__Patescibacteria;c__Paceibacteria;o__Moranbacterales;f__UBA2206;g__UBA11098;s__	
			2	d__Bacteria;p__Verrucomicrobiota;c__Omnitrophia;o__Omnitrophales	1
220926_sc-00148	85.34	82	1	d__Bacteria;p__Patescibacteria;c__ABY1;o__Magasanikbacterales;f__GWA2-37-8;g__;s__	
			2	d__Bacteria;p__Patescibacteria;c__Parcubacteria;o__Candidatus_Moranbacteria;f__Candidatus_Moranbacteria;g__Candidatus_Moranbacteria;s__	0.9988
			3	d__Bacteria;p__Proteobacteria;c__Gammaproteobacteria;o__Burkholderiales;f__Oxalobacteraceae;g__uncultured;s__	0.7486
220926_sc-00168	94.51	36.91	1	d__Bacteria;p__Patescibacteria;c__Paceibacteria;o__Moranbacterales;f__SCTJ01;g__;s__	
			2	d__Bacteria;p__Patescibacteria;c__Parcubacteria;o__Candidatus_Moranbacteria;f__Candidatus_Moranbacteria;g__Candidatus_Moranbacteria;s__	0.9997
220926_sc-00194	87.62	61.78	1	d__Bacteria;p__Patescibacteria;c__Paceibacteria;o__UBA9983_A;f__DSPV01;g__;s__	
			2	d__Archaea;p__Crenarchaeota;c__Nitrososphaeria;o__Nitrosotaleales;f__Nitrosotaleaceae;g__Candidatus_Nitrosotalea;s__	1
			3	d__Bacteria;p__Patescibacteria;c__Parcubacteria;o__Parcubacteria;f__Parcubacteria;g__Parcubacteria;s__	0.7087
			4	d__Bacteria;p__Proteobacteria;c__Gammaproteobacteria;o__Burkholderiales;f__Gallionellaceae;g__Candidatus_Nitrotoga;s__	0.977
			5	d__Bacteria;p__Proteobacteria;c__Gammaproteobacteria;o__Burkholderiales;f__Gallionellaceae;g__Candidatus_Nitrotoga;s__	0.8842
220926_sc-00244	70.53	40.55	1	d__Bacteria;p__Patescibacteria;c__Paceibacteria;o__UBA6257;f__Colwellbacteraceae;g__2-01-FULL-48-10;s__	
			2	d__Bacteria;p__Patescibacteria;c__Parcubacteria;o__Candidatus_Yanofskybacteria;f__Candidatus_Yanofskybacteria;g__Candidatus_Yanofskybacteria;s__	0.9999
			3	d__Bacteria;p__Patescibacteria;c__Parcubacteria;o__Candidatus_Jorgensenbacteria;f__Candidatus_Jorgensenbacteria;g__Candidatus_Jorgensenbacteria;s__	0.7469
220926_sc-00304	85.63	72.8	1	d__Bacteria;p__Patescibacteria;c__Paceibacteria;o__Paceibacterales;f__Staskawiczbacteraceae;g__;s__	
			2	d__Bacteria;p__Patescibacteria;c__Parcubacteria;o__Candidatus_Staskawiczbacteria;f__Candidatus_Staskawiczbacteria;g__Candidatus_Staskawiczbacteria;s__	0.9999
			3	d__Bacteria;p__Patescibacteria;c__Parcubacteria;o__Candidatus_Staskawiczbacteria;f__Candidatus_Staskawiczbacteria;g__Candidatus_Staskawiczbacteria;s__	1
			4	d__Bacteria;p__Patescibacteria;c__Parcubacteria;o__Parcubacteria;f__Parcubacteria;g__Parcubacteria;s__	1
220926_sc-00371	57.74	13.16	1	d__Bacteria;p__Patescibacteria;c__Paceibacteria;o__UBA9983_A;f__VMFH01;g__VMFH01;s__	
			2	d__Bacteria;p__Patescibacteria;c__Parcubacteria;o__Parcubacteria;f__Parcubacteria;g__Parcubacteria;s__	0.8503

For each SAG, the first entry (Detected 16S = 1) reports the genome-wide taxonomic classification assigned to the whole SAG assembly by GTDB-Tk. No Confidence value is given for this entry because GTDB-Tk placement is a phylogenomic classification, not a naive Bayes classification, and does not produce a directly comparable confidence score. Subsequent entries (Detected 16S = 2, 3, …) report the taxonomy independently assigned by SILVA 138.2 to each individual copy recovered from the assembly, together with the classifier’s confidence (0–1). Full results for all 46 CheckM-contaminated SAGs, including those not assigned to Patescibacteria, are provided in Supplementary Table S4.

## Data Availability

Raw sequencing reads generated in this study have been submitted to the DNA Data Bank of Japan (DDBJ) Sequence Read Archive (DRA) under BioProject accession PRJDB42729. Each of the 384 single-amplified genomes (SAGs) was registered as an individual BioSample (accession numbers SAMD01947188–SAMD01947454 and SAMD01949276–SAMD01949392) with a corresponding individual DRA Run accession (DRR1079629–DRR1080012); the full SAG-to-BioSample-to-Run correspondence is provided in Supplementary Table S1. Genome quality statistics, taxonomic assignments, geNomad predictions, and 16S rRNA classifications, KEGG pathway annotation outputs, COG functional category assignments, and pairwise average amino acid identity (AAI) matrices are provided in Supplementary Tables S1–S7, respectively.

## Supplementary Materials

The following supporting information can be downloaded at: https://www.mdpi.com/article/10.3390/microorganisms14102213/s1, Figure S1: presence and gene copy number of the 70 most broadly shared orthogroups across the 42 Patescibacteria SAGs; Table S1, genome quality, assembly statistics, sequencing read counts, GTDB-Tk taxonomic assignments, and corresponding DDBJ BioSample and DRA Run accession numbers for all 384 single-amplified genomes (SAGs); Table S2: genome quality metrics (CheckM and CheckM2), taxonomic classification, and phylogenomic tree membership of the 42 focal Patescibacteria SAGs; Table S3: genome-level summary of geNomad-predicted viral and plasmid sequences; Table S4: summary of the 46 SAGs classified as contaminated based on CheckM quality assessment, including SILVA taxonomic classification of the 18 individual 16S rRNA gene sequences recovered from 11 contaminated Patescibacteria SAGs; Table S5: KEGG Decoder pathway and module completeness outputs for the 42 focal Patescibacteria SAGs and reference genomes; Table S6: COG functional category assignments for the 42 focal Patescibacteria SAGs; Table S7: combined pairwise genome-similarity matrix showing average amino acid identity (AAI, %) below the diagonal and the number of reciprocal-best-hit (RBH) genes above the diagonal.
