# Supplementary material for: Single-Cell Genomics Uncovers an Unexpectedly Abundant, Phylogenetically Hyperdiverse Patescibacteria (Candidate Phyla Radiation) Community in Agricultural Paddy Soil
Source: Microorganisms. 2026 Oct 2;14(10):2213. doi: 10.3390/microorganisms14102213 (PMC13707452; doi:10.3390/microorganisms14102213)

Presence/ copy number of the 70 most broadly-shared orthogroups across 42 Patescibacteria SAGs

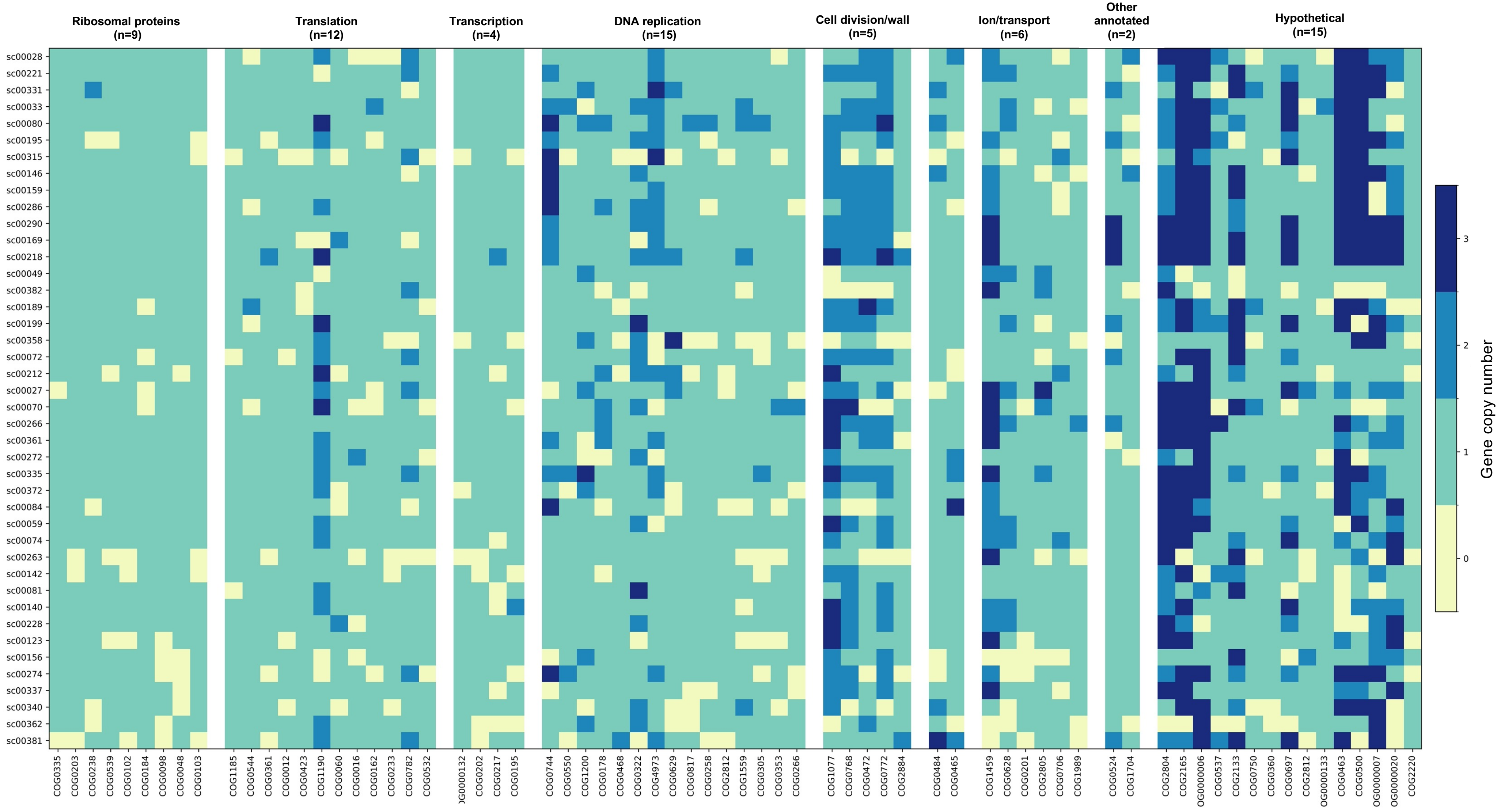

Supplement: Supplementary file 1 [file microorganisms-14-02213-s001.zip › Supplementry Figures_Patescibacteria2.pdf]
